# Dominant Role of Young’s Modulus for Electric Power Generation in PVDF–BaTiO_3_ Composite-Based Piezoelectric Nanogenerator

**DOI:** 10.3390/nano8100777

**Published:** 2018-09-30

**Authors:** Hyun Soo Kim, Dong Woo Lee, Do Hyung Kim, Dae Sol Kong, Jinhyeok Choi, Minbaek Lee, Gonzalo Murillo, Jong Hoon Jung

**Affiliations:** 1 Department of Physics, Inha University, Incheon 22212, Korea; star92123@naver.com (H.S.K.); 12131917@daum.net (D.W.L.); manwe2718@gmail.com (D.H.K.); lacam062@naver.com (D.S.K.); ch1j2005@naver.com (J.C.); mlee@inha.ac.kr (M.L.); 2 Department of Nano and Microsystems, Instituto de Microelectrónica de Barcelona (IMB-CNM, CSIC), 08193 Bellaterra, Spain; gonzalo.murillo@imb-cnm.csic.es

**Keywords:** piezoelectric nanogenerator, PVDF-BaTiO_3_ composite, Young’s modulus, dielectric constant

## Abstract

The electric power output of a piezoelectric nanogenerator (PENG) depends on the various physical parameters of the constituent materials, including the piezoelectric coefficient, Young’s modulus, and dielectric constant. Herein, we report the mechanical and electrical properties of a poly(vinylidene fluoride)–BaTiO_3_ (PVDF–BTO) composite-based PENG. Variation of the BTO nanoparticle (NP) content enabled the systematic tuning of the physical parameters that are related to power generation in the composite. The Young’s modulus of the PVDF–BTO composite initially increased, and then eventually decreased, with the increasing BTO content, which was probably due to the clustering effect of the high modulus BTO NPs. The dielectric constant of the composite continuously increased as the BaTiO_3_ content increased. The piezoelectric outputs were greatly enhanced at 10 wt% of BTO, where the Young’s modulus was the highest. These results indicate that the Young’s modulus plays an important role in the piezoelectric power generation of the composite-based PENGs.

## 1. Introduction

In recent years, there have been considerable interests in clean and renewable energies because of the rapid depletion of fossil resources and global warming [1]. Solar, wind, thermal, and vibrations are examples of renewable energies that can be converted into electricity [2,3]. Mechanical vibrational energy is abundant and ubiquitous, and depends less on environmental parameters such as time and geographical location than other types of energy [4,5]. Inorganic piezoelectric materials, such as Pb(Zr,Ti)O_3_, have been utilized to harvest high-frequency mechanical vibrations using resonant cantilever-type devices [6]. Inorganic and organic piezoelectric materials, such as ZnO and P(VDF–TrFE), have been utilized to harvest low-frequency mechanical vibrations using a piezoelectric nanogenerator (PENG) [7]. While the generated electricity is rather small, the greatly reduced power consumption of modern electronic devices enables a chance to realize self-powered devices without batteries. 

To effectively harvest random, low-frequency, and tiny mechanical vibrations in daily life, piezoelectric materials should be flexible and have large piezoelectric coefficients. Inorganic piezoelectric materials usually have large piezoelectric coefficients and Young’s moduli, but are fragile [8]. On the other hand, organic piezoelectric materials are flexible, but usually have small piezoelectric coefficients and Young’s moduli [9]. To balance these advantages and disadvantages, extensive research has been conducted to fabricate composite-type piezoelectric materials by blending two materials, e.g., PDMS–NaNbO_3_, PDMS–ZnSnO_3_, PMN–PT/PVDF, and PVDF–HFP/Co-ZnO [10,11,12,13]. These piezoelectric composites displayed piezoelectric outputs that were high enough to turn on the small electronic devices, such as light-emitting diodes (LEDs). To advance their applications, a systematic investigation of how changes in the physical properties affect the piezoelectric outputs of composite-based piezoelectric nanogenerators (PENGs) is highly required.

In this paper, we systematically investigate the mechanical and electrical properties, and piezoelectric power generation of poly(vinylidene fluoride)–BaTiO_3_ (PVDF–BTO) composites. Paraelectric BTO nanoparticles (NPs) do not affect the piezoelectric (ferroelectric) domains of PVDF during the electric poling process, but they do enable systematic variation of the physical properties. Since the Young’s modulus and dielectric constant of BTO are greater than those of PVDF, these properties increase with the increasing BTO content in the composite. Although the dielectric constant increases continuously, the Young’s modulus began to decrease above 10 wt% of the BTO. Intriguingly, the piezoelectric voltage and current were maximized at the 10 wt% level, at which the Young’s modulus was greatest. Additionally, the BTO wt%-dependent piezoelectric outputs predominantly followed the change in the Young’s modulus rather than the changes in the piezoelectric coefficient and dielectric constant. This work identifies a simple approach to increase the piezoelectric output of composite-based PENGs.

## 2. Experimental Details

### 2.1. Fabrication

**Figure 1:**
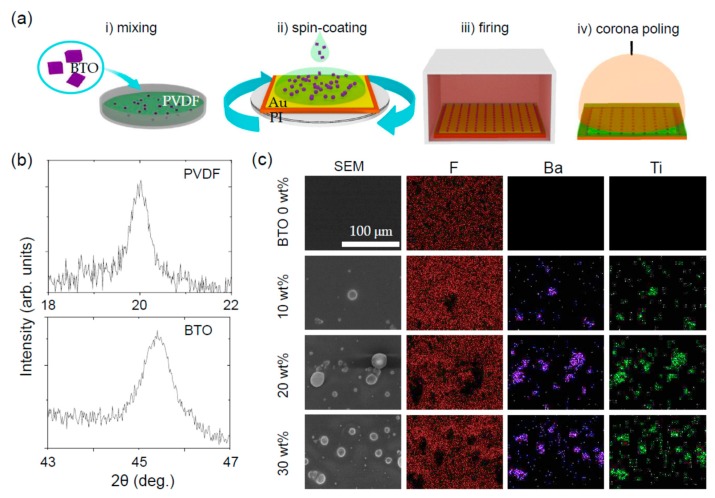
(**a**) Schematic diagram of the fabrication process of a poly(vinylidene fluorine)–BaTiO_3_ (PVDF–BTO) composite. (**i**) Mixing, (**ii**) spin-coating, (**iii**) firing, and (**iv**) coronal poling. (**b**) Magnified X-ray diffraction patterns at selected angles. (**c**) Scanning electron microscopy (SEM) images and energy-dispersive X-ray (EDX) spectral maps for F, Ba, and Ti atoms.

The PVDF–BTO composite films were prepared based on mixing, spin-coating, firing, and corona poling processes, as schematically shown in Figure 1a. Commercially available PVDF powder was dissolved in a dimethylformamide (DMF) solvent, and various amounts of BTO NPs were added to the PVDF solution. Ultrasonication was used to thoroughly mix and prevent the aggregation of the BTO NPs. A small aliquot of the PVDF–BTO solution was spin-coated on an Au-coated polyimide (PI) substrate (thickness ca. 150 μm) at the speed of 2000 rpm for 30 s. After the spin-coating, the PVDF–BTO solution was heated at 200 °C for 12 h to evaporate the solvent and crystallize. The PVDF–BTO film was corona-poled to align the ferroelectric, hence piezoelectric, domains in one direction. The corona poling was done by applying a high direct-current (DC) voltage (6 kV) to a needle that was 0.5 cm away from the PVDF–BTO film. The PVDF–BTO film was maintained at 100 °C, and the voltage was applied for 2 h. In contrast to conventional metal contact poling, the ionized particles in the air are accelerated and deposited on the composite during the coronal poling [14]. The ionized particles remain, and can stabilize the electric polarization of the composite based on Gross’s two-charge theory [15,16].

### 2.2. Characterization

The phase purity and crystalline quality of the PVDF–BTO composites were investigated by X-ray diffraction (XRD) using Cu Kα radiation. The surface profile and distribution of the BTO NPs in a composite were examined using a field-emission scanning electron microscope (FE-SEM) (S-4200, Hitachi, Tokyo, Japan) equipped with energy-dispersive X-ray (EDX) mapping. The Young’s modulus and dielectric constant were obtained using an atomic force microscope (AFM) and an LCR meter, respectively. 

### 2.3. Piezoelectric Power Generation Measurement

An Au-coated PI film electrode was attached to the upper surface of a corona-poled PVDF–BTO film. The thickness of the top electrode (ca. 75 μm) was less than that of the bottom PI film (ca. 125 μm). A PVDF–BTO composite-based PENG was mounted on a custom-designed mechanical bending system, in which a linear motor was used to periodically apply and release compressive forces to the PENG. The electrical outputs of the PENG were recorded by low-noise voltage and current preamplifiers. All of the electrical measurements were conducted in a Faraday cage to minimize noise.

### 2.4. Finite Element Computer Simulation

COMSOL Multiphysics software (Version 5.2a, COMSOL, Burlington, MA, USA) was used to simulate the strain in a PVDF–BTO composite. Due to computational limitations, only a small volume of ca. 3.375 μm^3^ was defined. The embedded BTO NPs (ca. 75 nm in diameter) were arranged in a regular array to emulate a perfectly mixed composite with an equivalent concentration of 10 wt%. The block was anchored on one side and subjected to a strain of 3.2% on the other side by means of a prescribed displacement. The bottom electrode was grounded, and the top electrode was considered a floating potential surface.

## 3. Results and Discussion

Figure 1b shows the X-ray diffraction (XRD) patterns of the PVDF–BTO composites. In particular, we magnified the XRD patterns for the ranges 18 ≤ 2θ ≤ 22 and 43 ≤ 2θ ≤ 47 to clarify the crystalline phases of the PVDF and BTO NPs. A sharp peak is evident near 2θ = 19.8°, which corresponds to the ferroelectric β-phase of PVDF [17]; the paraelectric α-phase of PVDF would show a peak at 2θ = 18.2°. The single sharp peak at 2θ = 45.4° corresponds to the paraelectric cubic phase of BTO [18]; the ferroelectric tetragonal phase of BTO would show split peaks. The paraelectric phase of BTO should be quite important because the ferroelectric, hence piezoelectric, domains of PVDF are not affected by the presence of BTO during the corona-poling process. The paraelectric phase of SrTiO_3_ should show a similar effect to BTO.

The distribution of the BTO NPs in the PVDF was examined using a scanning electron microscope (SEM) with energy dispersive X-ray (EDX) mapping capability. Figure 1c shows the BTO wt%-dependent surface morphology and distribution of F, Ba, and Ti atoms; the detailed EDX spectra are shown in Figure S1. The smooth PVDF morphology roughened with the increasing BTO content. Near the rough regions, the intensity of F atoms decreased, while the intensities of Ba and Ti atoms increased, which is consistent with the presence of BTO NPs. The SEM and EDX results indicate that the BTO NPs were well dispersed without significant aggregation. The BTO content of the PVDF–BTO composites that were obtained by analyzing their EDX spectra were in good agreement with the nominal values (Table 1). 

**Figure 2:**
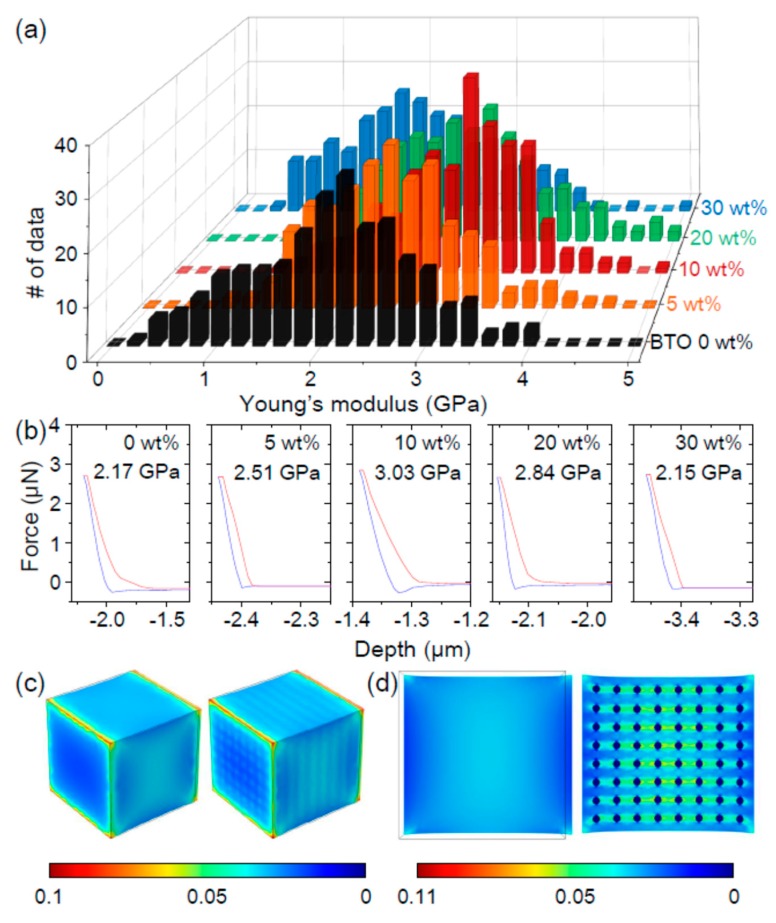
(**a**) Histogram of the Young’s modulus and (**b**) representative force–displacement curves. In (**b**), the red and blue lines represent increasing and decreasing applied force, respectively. Finite element simulations of the strain distribution (**c**) on the surface and (**d**) in the mid-layers of PVDF and PVDF–BTO blocks.

The mechanical properties of a PVDF–BTO composite were examined using the nanoindenter of atomic force microscopy (AFM) [19]. For statistical relevance, 256 different areas of the PVDF–BTO surface were measured. Figure 2a,b show a histogram of the Young’s modulus and force–displacement curves at selected moduli, respectively. Irrespective of BTO wt%, there was a large modulus variation, e.g., from 2.5 GPa to 3.2 GPa in PVDF. Additionally, there was a large displacement hysteresis with increasing (red lines) and decreasing (blue lines) applied force; the detailed force–displacement curves are shown in Figure S2. These behaviors should originate from the polymetric feature of the PVDF–BTO composite, as similarly observed in the PVDF–ZnO composite [20]. 

The average Young’s modulus that was measured for the PVDF was 2.17 GPa, which is in good agreement with the reported values [21]. The Young’s modulus of the composite initially increased with the increasing BTO content, because BTO has a higher modulus than PVDF [22]. Intriguingly, however, when the BTO content exceeded 10 wt%, the modulus decreased. Peng et al. recently reported the effect of the microstructure on the mechanical properties of nanocomposites [23]. Based on a numerical-analytical model, they showed that the Young’s modulus should decrease when the cluster size and number increase. As shown in Figure 1c, the BTO NPs seemed to aggregate to form sizable clusters. When the number of clusters increases, the modulus should initially increase and then decrease. The initial increase of the modulus should be more affected by the Young’s modulus of the BTO NPs, which is higher than that of the PVDF. The subsequent decrease of the modulus is thus attributed to increased numbers of BTO clusters.

The Young’s modulus should affect the strain of a material. To investigate the microscopic strain distribution in the PVDF–BTO composite, we used a COMSOL simulation (Figure 2c,d). The PVDF and BTO NPs were assumed to have rectangle and 75-nm diameter spherical shapes, respectively. When an external strain of 3.2% was applied along an axis, the PVDF–BTO composite showed a certain range of strain at the top surface, and a variation of strain from 1% to 11% in the mid-layer. On the other hand, the PVDF displayed a mean value of 3.2% of strain at the top surface and in the mid-layer, neglecting the higher values on the corners of the model, which could reach up to 4.3%. Due to the different Young’s modulus, a small strain occurs near BTO NPs and a large strain occurs near the PVDF. 

**Figure 3:**
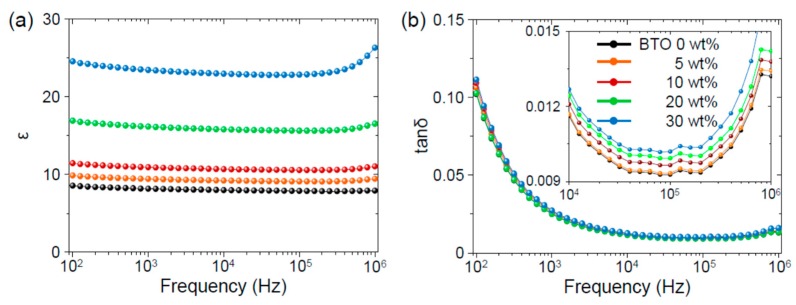
(**a**) Dielectric constants and (**b**) loss tangents of the PVDF–BTO composites. Magnified dielectric loss tangents are shown in the inset of (**b**).

The dielectric property of a PVDF–BTO composite was examined using an LCR meter over the frequency range of 10^2^–10^6^ Hz. Figure 3a,b show the dielectric constants and dielectric losses of the PVDF–BTO composites, respectively. The PVDF had a quite small dielectric constant and dielectric loss over a wide frequency range, as reported previously [24]. The dielectric constant systematically increased with increasing BTO content due to the large dielectric constant of BTO (ca. 1000 [25]). 

On the other hand, the dielectric loss increased only a little with increasing BTO content. The negligible dielectric loss (ca. 0.1), even at 30 wt% BTO, should be quite useful for the piezoelectric energy harvesting. A low dielectric loss should prevent the leakage of the piezoelectric surface charge and thereby increase the piezoelectric voltage and current [26]. 

**Figure 4:**
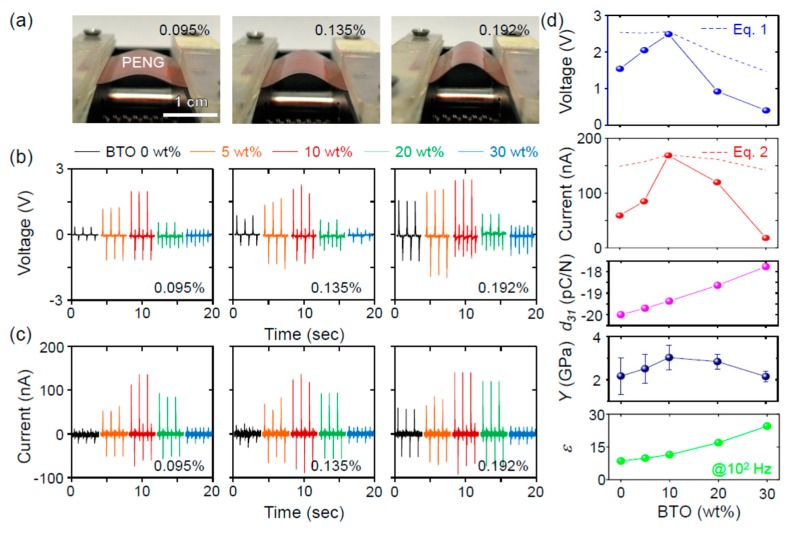
(**a**) Photographs of a bent PVDF–BTO composite piezoelectric nanogenerator (PENG) at selected strain values. The (**b**) open-circuit voltage and (**c**) closed-circuit current as a function of BTO content at selected strain values and at a fixed bending speed of 37 mm/s. (**d**) Comparison of the piezoelectric outputs with the predicted piezoelectric coefficient, measured Young’s modulus, and dielectric constant. The dashed lines in (**d**) represent the calculated piezoelectric outputs based on Equations (1) and (2).

From now on, we focus on the piezoelectric power generation of the PVDF–BTO composite-based piezoelectric nanogenerator (PENG). For systematic investigation, the device structure of the PENG does not change, except for the BTO content. Figure 4a shows the photographs of the PENG at various strains. The strain was quantified from the calculation of the strain neutral line and the lengths of bent PENG, as reported elsewhere [10,26]; the detailed calculation is provided in Figure S3. The calculated strain should be considered as an averaged value because of the distribution of strains in the composite. 

For the systematic investigation, the piezoelectric power outputs of PENGs were examined with different BTO contents, bending speeds, and load resistance (Figure S4). Figure 4b,c shows the open-circuit voltage and short-circuit current, respectively, of the PVDF–BTO composite-based PENGs at various strains. All of the devices showed enhanced voltage and current outputs at large strains. For all of the strains, on the other hand, the piezoelectric voltage and current initially increased, and then decreased as the BTO content increased. The piezoelectric outputs were maximized at 10 wt% of BTO. 

According to PENG theory [27], the piezoelectric voltage (*V*) and current (*I*) can be expressed as follows:

(1) $$\;V=\frac{d}{\varepsilon }\cdot Y\cdot t\cdot \frac{\cdot l}{l_{0}}\;$$

(2) $$\;I=\frac{d\cdot Y\cdot A}{l_{0}}\cdot \frac{d\cdot l}{dt}\;$$

where *d*, *Y*, *ε*, *A*, *t*, Δ*l*, and *l*_o_ represent the piezoelectric coefficient, Young’s modulus, dielectric constant, area, thickness, variation in length, and original length of the PENG, respectively. 

Figure 4d compares the piezoelectric outputs with the piezoelectric coefficient, Young’s modulus, and dielectric constant. We assumed the piezoelectric coefficient of the PVDF–BTO composite as the volume-weighted average of the two phases, i.e., *d*_31_ = *d*_PVDF_ × (1 − *x*) + *d*_BTO_ × *x*, where *d*_PVDF_ represents the piezoelectric coefficient of PVDF (ca. −21 pC/N [6,28]), *d*_BTO_ represents the piezoelectric coefficient of BTO NP (ca. 0 pC/N [29]), and *x* represents the volume percentage of BTO. The dependence of the piezoelectric output on the BTO content is more similar to the behavior of the Young’s modulus than to the trends in the piezoelectric coefficient and dielectric constant. 

In Figure 4d, we overlapped the piezoelectric outputs obtained from Equations (1) and (2). For direct comparison, the calculated voltage and current were normalized to the experimentally obtained ones at the BTO content of 10 wt%. It is evident that the calculated voltage and current are rather weakly dependent on the BTO content compared with the experimentally obtained values. This discrepancy is attributed to ignoring the distribution of Young’s moduli and the clustering effect on the piezoelectric coefficient. Considering the more polymetric features of the PVDF–BTO composite for lower BTO wt%, the Young’s modulus should be more distributed, which results in the distribution of piezoelectric outputs. Considering the piezoelectric coefficient of clustered BTO [29], the absolute value of the piezoelectric coefficient for PVDF–BTO should decrease, which eventually results in the further decreased piezoelectric outputs. 

## 4. Conclusions

In summary, we have investigated the piezoelectric power generation of the PVDF–BTO composite-based PENGs. The paraelectric BTO NPs enabled the systematic modification of the piezoelectric coefficient, Young’s modulus, and dielectric constant of the composite. The PVDF–BTO composite-based PENGs were fabricated using mixing, spin-coating, and poling processes on the Au-coated PI substrate. The statistically averaged Young’s modulus, which was obtained for 256 areas of the composite, initially increased and then decreased with the increasing BTO content. On the other hand, the dielectric constant continuously increased with a negligible change in dielectric loss. The piezoelectric voltage and current were maximized at the BTO content of 10 wt%, where the Young’s modulus was also maximized. Additionally, the piezoelectric voltage and current followed the same trend as the Young’s modulus. These results should indicate that the Young’s modulus significantly affects the electric power generation of composite-based PENGs.

## Figures and Tables

**Table 1:** BTO content of the PVDF–BTO composites obtained from their energy-dispersive X-ray (EDX) spectra.

BTO wt% (Nominal)	F wt%	Ba wt%	Ti wt%	BTO wt% (EDX)
0	64.67	1.41	0.49	2.15
10	63.36	2.83	0.98	4.60
20	56.27	9.45	3.28	14.46
30	49.91	14.51	5.04	22.631

## Supplementary Materials

The following are available online at http://www.mdpi.com/2079-4991/8/10/777/s1, Figure S1: energy-dispersive X-ray (EDX) spectra of the PVDF-BTO composite, Figure S2: detailed force-displacement curves at various BTO contents and Young’s modulus, Figure S3: schematic illustration of the strain calculation, Figure S4: bending speed and load-resistance dependent piezoelectric power.

## References

[1] Ginley D.S., Cahen D. (2011). Fundamentals of Materials for Energy and Environmental Sustainability.

[2] Dresselhaus M.S., Thomas I.L. (2001). Alternative energy technologies. Nature.

[3] Carrasco J.M., Bialasiewicz J.T., Guisado R.C.P., León J.I. (2006). Power-electronic systems for the grid integration of renewable energy sources: A survey. IEEE Trans. Ind. Electron..

[4] Wang Z.L., Song J.H. (2006). Piezoelectric nanogenerators based on zinc oxide nanowire arrays. Science.

[5] Fan F.R., Tian Z.Q., Wang Z.L. (2012). Flexible triboelectric generator. Nano Energy.

[6] Bowen C.R., Kim H.A., Weaver P.M., Dunn S. (2014). Piezoelectric and ferroelectric materials and structures for energy harvesting applications. Energy Environ. Sci..

[7] Wang Z.L., Wu W. (2012). Nanotechnology-enabled energy harvesting for self−powered micro-/nanosystems. Angew. Chem. Int. Ed..

[8] Park K.-I., Son J.H., Hwang G.-T., Jeong C.K., Ryu J., Koo M., Choi I., Lee S.H., Byun M., Wang Z.L. (2014). Highly-efficient, flexible piezoelectric PZT thin film nanogenerator on plastic substrates. Adv. Mater..

[9] Chang C., Tran V.H., Wang J., Fuh Y.-K., Lin W. (2010). Direct-write piezoelectric polymeric nanogenerator with high energy conversion efficiency. Nano Lett..

[10] Jung J.H., Lee M., Hong J.-I., Ding Y., Chen C.-Y., Chou L.-J., Wang Z.L. (2011). Lead-free NaNbO_3_ nanowires for a high output piezoelectric nanogenerator. ACS Nano.

[11] Lee K.Y., Kim D., Lee J.-H., Kim T.Y., Gupta M.K., Kim S.-W. (2014). Unidirectional high-power generation via stress-induced dipole alignment from ZnSnO_3_ nanocubes/polymer hybrid piezoelectric nanogenerator. Adv. Funct. Mater..

[12] Li C., Luo W., Liu X., Xu D., He K. (2016). PMN-PT/PVDF Nanocomposite for high output nanogenerator applications. Nanomaterials.

[13] Parangusan H., Ponnamma D., Al Ali Al-Maadeed M. (2018). Stretchable electrospun PVDF-HFP/Co-ZnO nanofibers as piezoelectric nanogenerators. Sci. Rep..

[14] Marshall J.M., Zhang Q., Whatmore R.W. (2008). Corona poling of highly (001)/(100)-oriented lead zirconate titanate thin films. Thin Solid Films.

[15] Gross B., de Moraes R.J. (1962). Polarization of the electret. J. Chem. Phys..

[16] Eisenmenger W., Schmidt H., Dehlen B. (1999). Space charge and dipoles in polyvinylideneuoride. Braz. J. Phys..

[17] Bellet-Amalric E., Legrand J.F. (1998). Crystalline structures and phase transition of the ferroelectric P(VDF-TrFE) copolymers, a neutron diffraction study. Eur. Phys. J. B.

[18] Kwon Y.H., Shin S.-H., Kim Y.-H., Jung J.-Y., Lee M.H., Nah J. (2016). Triboelectric contact surface charge modulation and piezoelectric charge inducement using polarized composite thin film for performance enhancement of triboelectric generators. Nano Energy.

[19] Tang B., Ngan A.H.W., Pethica J.B. (2008). A method to quantitatively measure the elastic modulus of materials in nanometer scale using atomic force microscopy. Nanotechnology.

[20] Choi M., Murillo G., Hwang S., Kim J.W., Jung J.H., Chen C.-Y., Lee M. (2017). Mechanical and electrical characterization of PVDF-ZnO hybrid structure for application to nanogenerator. Nano Energy.

[21] Ramadan K.S., Sameoto D., Evoy S. (2014). A review of piezoelectric polymers as functional materials for electromechanical transducers. Smart Mater. Struct..

[22] Park K.-I., Lee M., Liu Y., Moon S., Hwang G.-T., Zhu G., Kim J.E., Kim S.O., Kim D.K., Wang Z.L. (2012). Flexible nanocomposite generator made of BaTiO_3_ nanoparticles and graphitic carbons. Adv. Mater..

[23] Peng R.D., Zhou H.W., Wang H.W., Mishnaevsky L. (2012). Modeling of nano-reinforced polymer composites: Microstructure effect on Young’s modulus. Comp. Mater. Sci..

[24] Fu J., Hou Y., Zheng M., Wei Q., Zhu M., Yan H. (2015). Improving dielectric properties of PVDF composites by employing surface modified strong polarized BaTiO_3_ particles derived by molten salt method. ACS Appl. Mater. Interfaces.

[25] Wang X.-H., Chen I.-W., Deng X.-Y., Wang Y.-D., Li L.-T. (2015). New progress in development of ferroelectric and piezoelectric Nanoceramics. J. Adv. Ceram..

[26] Jung J.H., Chen C.-Y., Yun B.K., Lee N., Zhou Y., Jo W., Chou L.-J., Wang Z.L. (2012). Lead-free KNbO_3_ ferroelectric nanorods based flexible nanogenerators and capacitors. Nanotechnology.

[27] Jaffe B., Cook W.R., Jaffe H. (1971). Piezoelectric Ceramics.

[28] Katsouras I., Asadi K., Li M., van Driel T.B., Kjaer K.S., Zhao D., Lenz T., Gu Y., Blom P.W.M., Damjanovic D. (2016). The negative piezoelectric effect of the ferroelectric polymer poly(vinylidene fluoride). Nature.

[29] Hoshina T. (2013). Size effect of barium titanate: Fine particles and ceramics. J. Ceram. Soc. Jpn..

