# Supplementary material for: Dominant Role of Young’s Modulus for Electric Power Generation in PVDF–BaTiO3 Composite-Based Piezoelectric Nanogenerator
Source: Nanomaterials (Basel). 2018 Sep 30;8(10):777. doi: 10.3390/nano8100777 (PMC6215192; doi:10.3390/nano8100777)
Supplement: Supplementary file 1 [file nanomaterials-08-00777-s001.pdf]

Supplementary Materials

Dominant Role of Young′s Modulus for Electric Power Generation in PVDF-$BaTiO_3$ Composite-based Piezoelectric Nanogenerator

Hyun Soo Kim[1], Dong Woo Lee[1], Do Hyung Kim[1], Dae Sol Kong[1], Jinhyeok Choi[1], Minbaek Lee[1], Gonzalo Murillo[2] and Jong Hoon Jung[1,*]

[1]Department of Physics, Inha University, Incheon 22212, Republic of Korea

[2]Department of Nano and Microsystems, Instituto de Microelectrónica de Barcelona (IMB-CNM, CSIC), Bellaterra 08193, Spain

[*]Corresponding author

Email: jhjung@inha.ac.kr;

Tel: +82-32-860-7659;

Fax: +82-32-872-7562

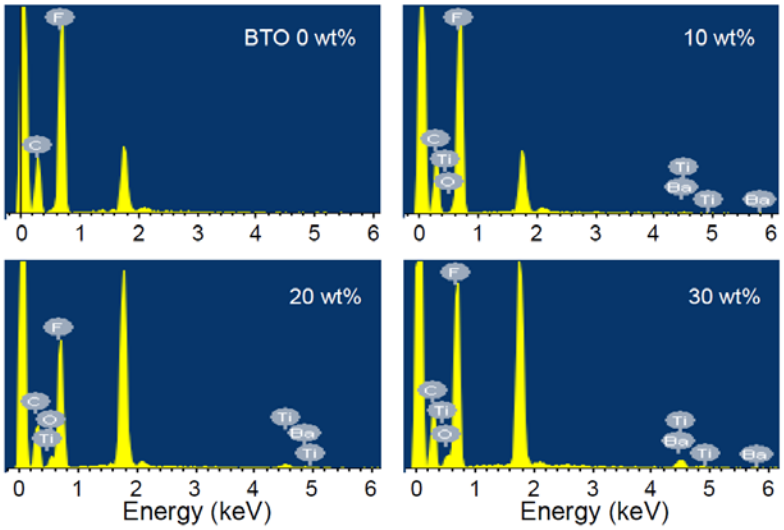

Figure S1. Energy-dispersive X-ray (EDX) spectra of the poly(vinylidene fluoride)–$BaTiO_3$ (PVDF-BTO) composite.

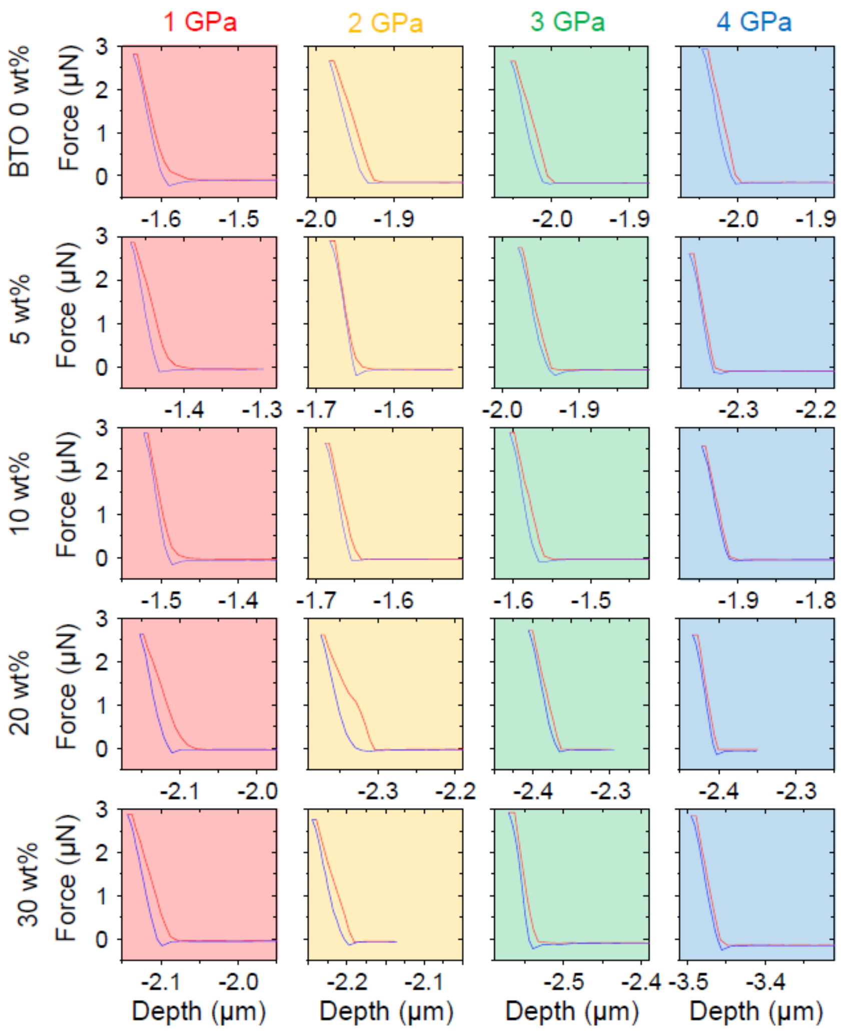

Figure S2. Detailed force–displacement curves at various BTO contents and Young’s modulus.

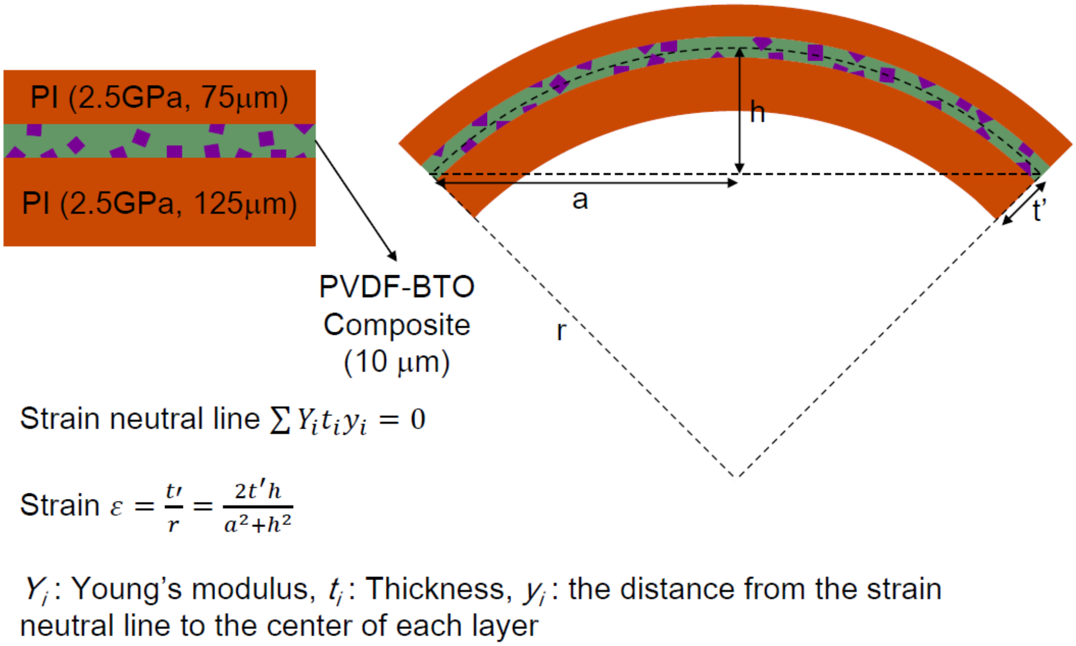

Figure S3. Schematic illustration of the strain calculation.

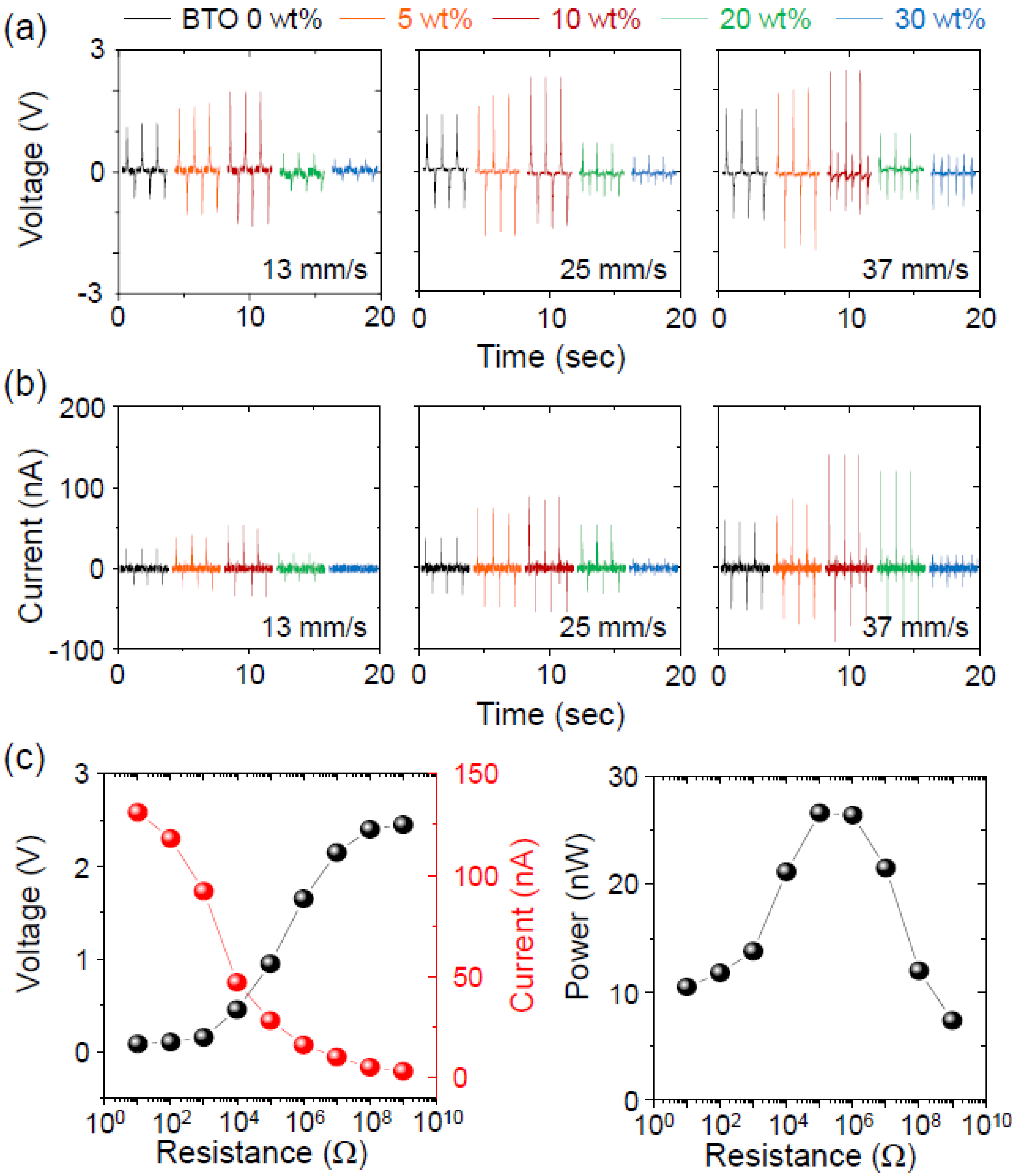

Figure S4. (**a**) Open-circuit voltage and (**b**) closed-circuit current of the PVDF–BTO composite based PENG as a function of BTO content and bending speed. (**c**) Load-resistance dependent piezoelectric power. The maximum power outputs occur at the load resistance of 1 MΩ.
